# The prevalence of shoulders with a large glenoid defect and small bone fragment increases after several instability events during conservative treatment for traumatic anterior instability

**DOI:** 10.1016/j.jseint.2023.03.008

**Published:** 2023-04-10

**Authors:** Shigeto Nakagawa, Yasuhiro Take, Naoko Mizuno, Ritsuro Ozaki, Hiroto Hanai, Ryo Iuchi, Kazutaka Kinugasa

**Affiliations:** a Department of Orthopaedic Sports Medicine, Yukioka Hospital, Osaka, Osaka, Japan; b Department of Orthopaedic Surgery, Daini Police Hospital, Osaka, Osaka, Japan; c Department of Orthopaedic Surgery, Toyonaka Municipal Hospital, Toyonaka, Osaka, Japan; d Sanjodori Orthopaedic Clinic, Nara, Nara, Japan; e Department of Orthopaedic Surgery, Osaka University, Graduate School of Medicine, Suita, Osaka, Japan; f Department of Orthopaedic Surgery, Seihu Hospital, Sakai, Osaka, Japan; g Department of Orthopaedic Sports Medicine, Osaka Rosai Hospital, Sakai, Osaka, Japan

**Keywords:** Traumatic anterior shoulder instability, Conservative treatment, Glenoid defect size, Bone fragment size, Subcritical glenoid defect, Glenoid defect enlargement

## Abstract

**Background:**

Unstable shoulders with a large glenoid defect and small bone fragment are at higher risk for postoperative recurrence after arthroscopic Bankart repair. The purpose of the present study was to clarify the changes in the prevalence of such shoulders during conservative treatment for traumatic anterior instability.

**Methods:**

We retrospectively investigated 114 shoulders that underwent conservative treatment and computed tomography (CT) examination at least twice after an instability event in the period from July 2004 to December 2021. We investigated the changes in glenoid rim morphology, glenoid defect size, and bone fragment size from the first to the final CT.

**Results:**

At first CT, 51 shoulders showed no glenoid bone defect, 12 showed glenoid erosion, and 51 showed a glenoid bone fragment [33 small bone fragment (<7.5%) and 18 large bone fragment (≥7.5%); mean size: 4.9 ± 4.2% (0-17.9%)]. Among patients with glenoid defect (fragment and erosion), the mean glenoid defect was 5.4 ± 6.6% (0-26.6%); 49 were considered a small glenoid defect (<13.5%) and 14 were a large glenoid defect (≥13.5%). While all 14 shoulders with large glenoid defect had a bone fragment, small fragment was solely seen in 4 shoulders. At final CT, 23 of the 51 shoulders persisted without glenoid defect. The number of shoulders presenting glenoid erosion increased from 12 to 24, and the number of shoulders with bone fragment increased from 51 to 67 [36 small bone fragment and 31 large bone fragment; mean size: 5.1 ± 4.9% (0-21.1%)]. The prevalence of shoulders with no or a small bone fragment did not increase from first CT (71.4%) to final CT (65.9%; *P* = .488), and the bone fragment size did not decrease (*P* = .753). The number of shoulders with glenoid defect increased from 63 to 91 and the mean glenoid defect significantly increased to 9.9 ± 6.6% (0-28.4%) (*P* < .001). The number of shoulders with large glenoid defect increased from 14 to 42 (*P* < .001). Of these 42 shoulders, 19 had no or a small bone fragment. Accordingly, among a total of 114 shoulders, the increase from first to final CT in the prevalence of a large glenoid defect accompanied by no or a small bone fragment was significant [4 shoulders (3.5%) vs. 19 shoulders (16.7%); *P* = .002].

**Conclusions:**

The prevalence of shoulders with a large glenoid defect and small bone fragment increases significantly after several instability events.

Sugaya et al investigated the anterior glenoid rim morphologic characteristics using 3-dimensional reconstructed computed tomography (3D-CT) in shoulders with recurrent anterior instability and found fragment-type glenoid defect in 50% of the shoulders and erosion-type glenoid defect in 40% of them.^19^ Burkhart and De Beer reported that patients with an inverted pear glenoid were not candidates for arthroscopic Bankart repair (ABR).^1^ The critical glenoid defect size was suggested to be 20% to 25%.^6^^,^^9^^,^^20^ On the other hand, Shaha et al reported that an appropriate threshold for ‘‘subcritical’’ glenoid defect in active duty military personnel was 13.5%, which led to a clinically significant worsening in Western Ontario Shoulder Instability Index scores.^18^ Recently, a glenoid defect of 13.5% or more is the most popular value as the definition of a subcritical glenoid defect.^2^^,^^5^^,^^7^^,^^8^^,^^21^

In patients with such a large glenoid defect and a bone fragment, at recurrent anterior instability the bone fragment sometimes appears small compared with the size of the glenoid defect (Fig. 1). In a study on small bone fragments in patients with bony Bankart lesions, Nakagawa et al reported that most bone fragments undergo extensive resorption in the first year after the primary trauma.^15^ In contrast, the bone fragment may appear smaller not because of resorption but because the glenoid defect itself has become larger as a result of damage due to repetitive instability events.^4^^,^^10^^,^^17^ Nakagawa et al showed that if the residual bone fragment was small at arthroscopic bony Bankart repair (ABBR), the postoperative bone union rate was lower, bone union was delayed, and the postoperative recurrence rate was higher.^14^^,^^16^ Consequently, they suggested that it is important to repair a bony Bankart lesion as soon as possible before the bone fragment size decreases.

**Figure 1:**
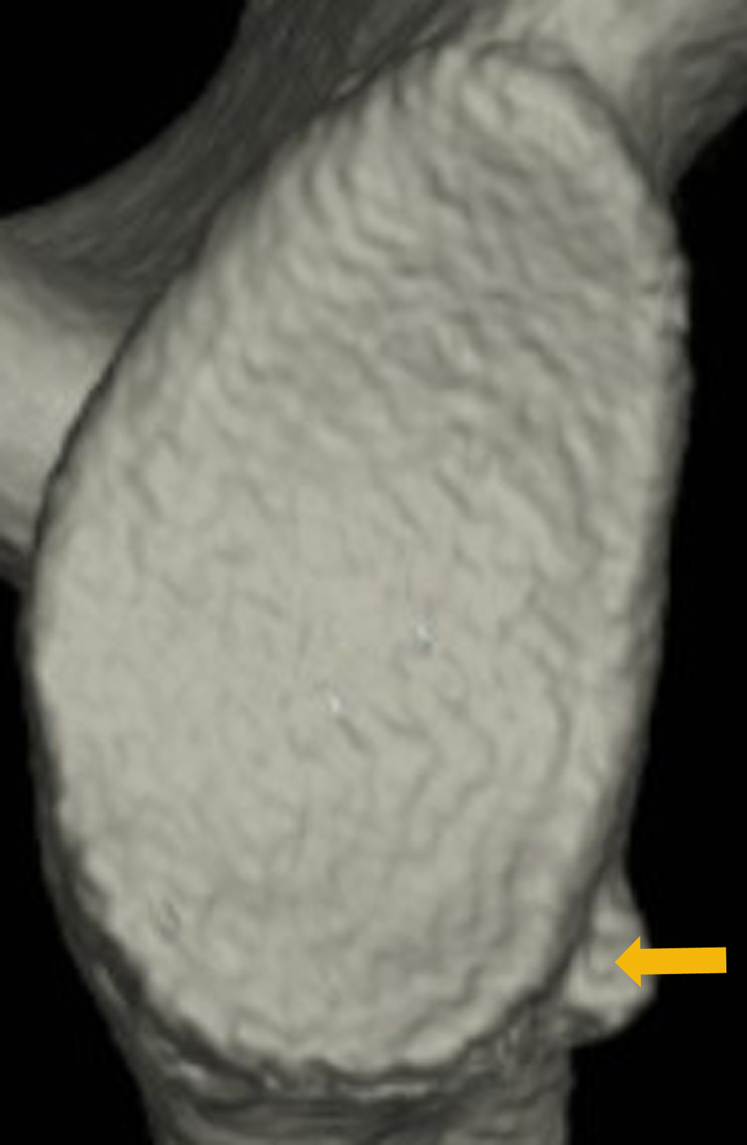
The mismatch between glenoid defect size and bone fragment size in a shoulder with recurrent anterior instability. A bone fragment appears small compared with the size of the glenoid defect (: bone fragment) (Right shoulder).

Nakagawa et al recently reported that the postoperative recurrence rate after ABBR was lower in shoulders with a larger glenoid defect (≥ 13.5%) than in shoulders with a smaller glenoid defect (< 13.5%) even in male competitive rugby and American football players.^12^ As a large bone fragment (≥ 7.5%) frequently remained in shoulders with a large glenoid defect, the rate of complete bone union was significantly higher in shoulders with a large bone fragment than in shoulders with a small bone fragment (< 7.5%). They concluded that the postoperative recurrence rate was significantly lower after complete bone union, so the presence of a large bone fragment might decrease the recurrence rate in shoulders with a large glenoid defect. In other words, if the bone fragment was smaller than 7.5%, shoulders with a large glenoid defect (≥ 13.5%) appeared to be at higher risk for postoperative recurrence after ABBR. However, the reason for this apparent association between glenoid defect size and bone fragment size is unclear.

In the present study, we evaluated shoulders that underwent serial CT examination during conservative treatment after traumatic anterior instability. We investigated the changes in glenoid defect and bone fragment size from the first to the final CT with the aim to clarify the changes in the prevalence of shoulders with a large glenoid defect and small bone fragment during conservative treatment for traumatic anterior instability. We hypothesized that the prevalence of such shoulders increases after several instability events.

## Materials and methods

This was a retrospective case series study. The study was approved by the local institutional review board, and participants gave a written informed consent to participate.

Patients who underwent conservative treatment after traumatic anterior shoulder instability (not restricted to patients with primary instability) and experienced further recurrence of instability in absence of stabilization surgery in the period from July 2004 to December 2021 were included in this study. They also underwent serial CT examinations at least twice after an instability event, which were searched through the hospital imaging system. First CT examination was performed at the first hospital visit and second or third CT examination was performed after further recurrence of instability. Patients whose instability recurred after the first CT (> 6 months) were again evaluated by CT, but to decrease radiation exposure, CT evaluation was usually not performed in patients whose instability recurred within 6 months and magnetic resonance imaging evaluation was performed alternatively. Exclusion criteria were patients with previous stabilization surgery and those with less than 2 CT scans.

Among 1268 shoulders with traumatic anterior instability from July 2004 to December 2021, 78 shoulders were ineligible for inclusion because they had undergone previous stabilization surgery and 1076 shoulders because no or only 1 CT examination was performed. The remaining 114 shoulders that underwent conservative treatment and serial CT examination at least twice (after an instability event and after further recurrence of instability) were included in the study (13 patients were affected bilaterally). CT examination was performed twice in 105 shoulders and 3 times in 9 shoulders. The demographics of the included shoulders are shown in Table I. Stabilization surgery was performed after final CT in 87 shoulders.

**Table I:** Demographics.

	Timing of CT	Glenoid rim morphology at first CT		
		Normal	Erosion type	Fragment type
No. of shoulders		51	12	51

Gender				
Male		43 (84.3%)	8 (66.7%)	44 (86.3%)
Female		8	4	7
Sports				
Rugby		13 (25.5%)	1 (8.3%)	17 (33.3%)
Am. football		12 (23.5%)	1 (8.3%)	11 (21.6%)
Collision/contact		11 (21.6%)	8 (66.7%)	15 (29.4%)
Overhead		11 (21.6%)	0 (0%)	1 (2.0%)
Others		2	0	1
Recreational		1	0	3
No sports		1	2	3
Age at primary instability		18.1 ± 5.5 yrs	20.3 ± 6.5 yrs	19.6 ± 9.3 yrs
		(13-34 yrs)	(13-34 yrs)	(12-74 yrs)
Age at CT	First CT	18.4 ± 5.5 yrs	23.7 ± 7.4 yrs	21.4 ± 9.8 yrs
		(13-44 yrs)	(16-40 yrs)	(13-74 yrs)
	Final CT	19.6 ± 5.6 yrs	25.4 ± 7.9 yrs	22.9 ± 10.2 yrs
		(15-45 yrs)	(17-42 yrs)	(14-77 yrs)
Period since primary instability	First CT	0.3 ± 0.7 yrs	3.3 ± 4.0 yrs	1.6 ± 3.7 yrs
		(0-4 yrs)	(0-13 yrs)	(0-23 yrs)
	Final CT	1.3 ± 1.5 yrs	5.0 ± 3.9 yrs	3.0 ± 4.5 yrs
		(0-6 yrs)	(0-14 yrs)	(0-28 yrs)
Total instability events	First CT	2.0 ± 4.2	6.6 ± 7.1	10.4 ± 22.5
		(1-30)	(1-24)	(1-120)
	Final CT	5.6 ± 8.0	16.8 ± 18.1	19.5 ± 31.1
		(2-40)	(4-65)	(2-150)
Glenoid defect size	First CT	0.0 ± 0.0%	7.1 ± 3.6%	10.3 ± 6.4%
		(0.0%)	(2.7-10.0%)	(0.8-26.6%)
	Final CT	4.7 ± 5.9%	12.0 ± 4.0%	14.6 ± 6.5%
		(0.0-25.8%)	(5.1-18.1%)	(1.8-28.4%)
Bone fragment size	First CT	N/A	0.0 ± 0.0%	6.0 ± 3.9%
			(0.0%)	(0.4-17.9%)
	Final CT	2.8 ± 3.6%	1.5 ± 3.0% (0.0-9.0%)	7.3 ± 4.8% (0.0-21.1%)
		(0.0-11.1%)		
		(in 28 shoulders with a glenoid defect)		

*CT*, computed tomography.

CT scanning and reconstruction of images (spiral scan, 0.5-mm slice thickness, 0.3-mm reconstruction, and 3D edit mode) were performed, and to perform multiplanar reconstruction, CT data were analyzed in Digital Imaging and Communications in Medicine mode with Digital Imaging and Communications in Medicine software.

**Figure 2:**
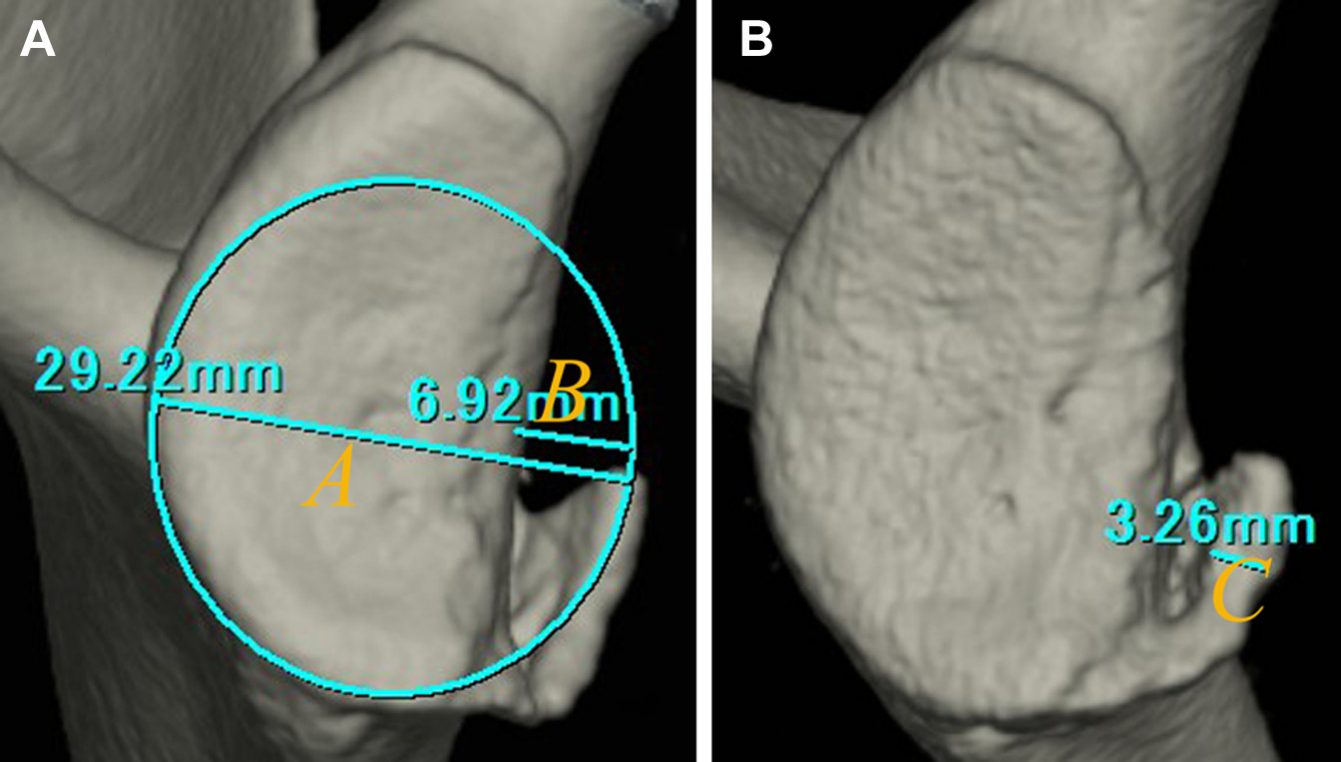
Quantification method for the glenoid defect size and bone fragment size: The glenoid defect size: *B/A* × 100%: The bone fragment size: *C*/*A* × 100%. (**A**), the glenoid rim width; (**B**), the width of the glenoid defect; (**C**), the width of the bone fragment.

The classification of glenoid rim morphology by Sugaya et al was used to divide into 3 groups: normal, erosion, and fragment type. To measure the glenoid defect size and bone fragment size, after elimination of the humeral head, the inferior portion of the glenoid face was approximated to a true circle on face 3D-CT scans. The glenoid rim width (*A*) and width of the glenoid defect (*B*) were measured, and in accordance with the method by Nakagawa et al,^14,16^ the width of the bone fragment (*C*) was measured on the CT image that gave the clearest view of the articular surface. The extent of the glenoid defect was calculated as a percentage of the glenoid rim width (glenoid defect size: *B/A* × 100%), and the extent of the bone fragment measured at the widest portion was defined relative to the glenoid rim width (bone fragment size: *C*/*A* × 100%) (Fig. 2). We determined the intraobserver reliability by comparing 2 intraobserver measurements performed 1 month apart, and the interobserver reliability was determined by comparing measurements by 2 independent observers.

We investigated glenoid rim morphology, glenoid defect size, and bone fragment size at first CT. Then, the changes from the first to the final CT were investigated. For patients who underwent CT twice, glenoid defect size and bone fragment size at the second CT were evaluated. For patients who underwent CT 3 times, the evaluation was performed at the third CT. If the glenoid defect was smaller than 13.5%, it was categorized as “small,” and if it was 13.5% or larger, it was categorized as “large.” If the bone fragment was smaller than 7.5%, it was categorized as “small,” and if it was 7.5% or larger, it was categorized as “large,” as per Nakagawa et al’s report.^12^

### Statistical analysis

Mean values were compared between 2 groups with Student’s *t*-test or Mann-Whitney U test. The ratios were compared by Fisher’s exact probability test. When the *P* value was less than .05, a post hoc power analysis was performed. If the study had sufficient statistical power (1-β ≥ 0.8), we determined the difference to be significant.

To investigate the intraobserver and interobserver reliabilities of measurements of the glenoid defect and bone fragment size, we determined the intraclass correlation coefficient (1, 1) and interclass correlation coefficient (2, 1); a value > 0.8 was judged as good reliability.

## Results

### Glenoid rim morphology at first computed tomography

At first CT, 51 shoulders showed no glenoid bone defect, 12 showed glenoid erosion, and 51 showed a glenoid bone fragment [33 small bone fragment (< 7.5%) and 18 large bone fragment (≥ 7.5%); mean size: 4.9 ± 4.2% (0-17.9%)] (Table II). Between patients with glenoid defect (fragment and erosion), the mean glenoid defect was 5.4 ± 6.6% (0-26.6%); 49 were considered a small glenoid defect (< 13.5%) and 14 were a large glenoid defect (≥13.5%) (Table III). While all 14 shoulders with large glenoid defect had a bone fragment, small fragment was solely seen in 4 shoulders (Table IV).

**Table II:** The changes in bone fragment size from first to final CT.

Glenoid rim morphology at first CT	Bone fragment size		No. of patients
	First CT	Final CT	
Normal		no	13
		small	10
		large	5
Erosion	No	no	9
	No	small	2
	No	large	1
Fragment	Small	no	2
	Small	small	23
	Small	large	8
	Large	small	1
	Large	large	17

*CT*, computed tomography.

**Table III:** The changes in glenoid defect size from first to final CT.

Glenoid rim morphology at first CT	Glenoid defect size		No. of patients
	First CT	Final CT	
Normal	no	no	23
	no	small	23
	no	large	5
Erosion	small	small	7
	small	large	5
Fragment	small	small	19
	Small	large	18
	Large	large	14

*CT*, computed tomography.

**Table IV:** Bone fragment size in shoulders with a large glenoid defect of 13.5% or larger at first and final CT.

Glenoid rim morphology at first CT	Timing of CT	Total	Bone fragment size		
			No	Small	Large
Normal	first CT	0	0	0	0
	final CT	5	2	2	1
Erosion	first CT	0	0	0	0
	final CT	5	3	1	1
Fragment	first CT	14	0	4	10
	final CT	32	1	10	21
Total	first CT	14	0	4	10
	final CT	42	6	13	23

The intraobserver reliabilities of glenoid defect size and bone fragment size were 0.985 and 0.953 and the interobserver reliabilities were 0.914 and 0.845, respectively.

### Changes in glenoid rim morphology from first to final computed tomography

At final CT, 23 of the 51 shoulders persisted without glenoid defect. The number of shoulders presenting glenoid erosion increased from 12 to 24, and the number of shoulders with bone fragment increased from 51 to 67 [36 small bone fragment and 31 large bone fragment; mean size: 5.1 ± 4.9% (0-21.1%)]. The prevalence of shoulders with no or a small bone fragment did not increase from first CT (71.4%) to final CT (65.9%; *P* = .488), and the bone fragment size did not decrease (*P* = .753) (Table II).

The number of shoulders with glenoid defect increased from 63 to 91 and the mean glenoid defect significantly increased to 9.9 ± 6.6% (0-28.4%) (*P* < .001). The number of shoulders with large glenoid defect increased from 14 to 42 (*P* < .001) (Table III).

Of these 42 shoulders, 19 had no or a small bone fragment (Table IV). Accordingly, among a total of 114 shoulders, the increase from first to final CT in the prevalence of a large glenoid defect accompanied by no or a small bone fragment was significant [4 shoulders (3.5%) vs. 19 shoulders (16.7%); *P* = .002] (Table IV).

### Change in bone fragment size relative to change in glenoid defect size in shoulders with fragment-type morphology at first CT

In shoulders with a large glenoid defect and a small bone fragment at first CT (n = 4), the bone fragment was large in 2 shoulders but still small in the other 2 shoulders at final CT. In shoulders with a small glenoid defect and small bone fragment at first CT and a large glenoid defect at final CT (n = 14), the bone fragment was large in 5 shoulders, small in 8 shoulders, and no longer present in 1 shoulder at final CT. Thus, no or a small bone fragment with a large glenoid defect at final CT was seen in 11 (21.6%) of 51 shoulders with fragment-type glenoid rim morphology at first CT (Table V).

**Table V:** The change in bone fragment size relative to change in glenoid defect size in shoulders with a fragment-type morphology at first CT.

From first to final CT	Change in glenoid defect size			Total
	Small to small	Small to large	Large to large	
Change in bone fragment size				
Small to no	1	1	0	2
Small to small	13	8	2	23
Small to large	1	5	2	8
Large to small	1	0	0	1
Large to large	3	4	10	17
Total	19	18	14	51

## Discussion

The important finding in the present study was that in shoulders that underwent conservative treatment for traumatic anterior instability and CT examination at least twice after an instability event, the prevalence of shoulders with a large glenoid defect (≥ 13.5%) accompanying a small bone fragment (<7.5%) increased significantly after several instability events, and this increase was due to enlargement of the glenoid defect.

Nakagawa et al reported that resorption of bone fragments progresses soon after the primary instability event.^15^ However, in the present study, bone fragment resorption was quite rare. Although a small bone fragment at first CT sometimes became large at final CT, bone fragment size was usually unchanged from the first to the final CT. Thus, if a small bone fragment is found at first CT, it may still be small at final CT. The present study showed a significant increase in the size of glenoid defects from first to final CT, which was consistent with several previous reports.^4^^,^^10^^,^^17^ Accordingly, this study provides further evidence that the prevalence of shoulders with a large glenoid defect and small bone fragment increases after several instability events because of enlargement of the glenoid defect.

The results on glenoid rim morphology showed that in more than half of the shoulders, glenoid morphology changed from normal at first CT to erosion or fragment type at final CT. Among the 51 shoulders with a normal glenoid at first CT, a large glenoid defect was found in 5 shoulders (9.8%) and no or a small bone fragment was seen in 4 (80%) of 5 shoulders at final CT. Among the 12 shoulders with erosion-type glenoid rim morphology at first CT, a large glenoid defect was found in 5 shoulders (41.7%) and no or a small bone fragment was seen in 4 (80%) of 5 shoulders at final CT. On the other hand, among the 51 shoulders with fragment-type glenoid rim morphology at first CT, the prevalence of a large glenoid defect and no or a small bone fragment increased from 4 shoulders (7.8%) at first CT to 11 shoulders (21.6%) at final CT. Of note, in the 18 shoulders with a small glenoid defect at first CT and a large glenoid defect at final CT, 9 (50%) had no or a small bone fragment at final CT. Among all the shoulders with a large glenoid defect, at first CT no or a small bone fragment was present in 4 shoulders (28.6%) and a large bone fragment was present in 10 shoulders (71.4%), but the respective numbers at final CT were 19 (45.2%) and 23 (54.8%). Thus, the prevalence of shoulders with a large glenoid defect and no or a small bone fragment increased from the first to the final CT in all types of glenoid rim morphology at first CT.

The present study also found that shoulders with a large glenoid defect had a large bone fragment more often than no or a small fragment at the first and final CT. This result is compatible with a recent study by Nakagawa et al, which showed that at recurrent instability a larger bone fragment frequently remains in shoulders with a large glenoid defect.^11^ Nakagawa et al also reported that even in younger competitive athletes, postoperative recurrence rate after ABBR was lower when a remaining larger bone fragment was repaired.^12^ As in the present study, not only was a large bone fragment at first CT often still large at final CT but a small bone fragment at first CT sometimes became large at final CT. This finding indicates that even in shoulders with a large glenoid defect at final CT, the prevalence and size of remaining bone fragments should be carefully evaluated.

Accordingly, when planning surgery for traumatic anterior shoulder instability, patients should be categorized as per the glenoid rim morphology at first CT, that is, into normal or large fragment type and erosion or small fragment type. Bone union and postoperative recurrence rates are known to be significantly different between shoulders with a large bone fragment and those with a small bone fragment. Nakagawa et al reported that the postoperative recurrence rate after ABR was higher in shoulders with a small bone fragment and was similar to the rate in shoulders without a bone fragment.^13^ Furthermore, Nakagawa et al also reported that nonunion or disappearance of a small bone fragment was often seen after repair of a small bone fragment.^16^ Recently, Di Giacomo et al also found a significant inverse relationship between preoperative bone fragment size and the percentage of postoperative resorption and concluded that a smaller fragment size can result in worse clinical outcomes because of resorption.^3^ Consequently, even if a glenoid defect is small, early stabilization surgery might be indicated for shoulders with no or a small bone fragment before the glenoid defect becomes larger. On the other hand, Nakagawa et al reported that the postoperative recurrence rate was not as high when a remaining large bone fragment was repaired at ABR.^11^^,^^12^ In shoulders with a large bone fragment, the rate of bone union after ABBR is high even in cases of chronic instability, and glenoid rim morphology is expected to normalize because of remodeling of the united bone fragment.^14^ Thus, early stabilization surgery might not be always required.

### Limitations

In the present study, there were some limitations, including the retrospective design. Although we compared glenoid rim morphology at first and final CT, the timing of these CTs was different among the 3 groups classified by glenoid rim morphology. Furthermore, this retrospective study did not compare CT findings at the primary instability and recurrence, so this should be prospectively evaluated in a future study. Finally, Hill-Sachs defect has not been measured in the present study, which was well known to affect clinical outcomes after stabilization surgery.

## Conclusion

The prevalence of shoulders with a large glenoid defect (≥ 13.5%) and small bone fragment (< 7.5%) increases significantly after several instability events.
